# Ischemic stroke: experimental models and reality

**DOI:** 10.1007/s00401-017-1667-0

**Published:** 2017-01-07

**Authors:** Clemens J. Sommer

**Affiliations:** Institute of Neuropathology, University Medical Center of the Johannes Gutenberg-University Mainz; Focus Program Translational Neuroscience (FTN) and Rhine Main Neuroscience Network (rmn2), Langenbeckstrasse 1, 55131 Mainz, Germany

**Keywords:** Animal model, Cerebral ischemia, Focal ischemia, In vitro model, Non-human primate, Stroke

## Abstract

The vast majority of cerebral stroke cases are caused by transient or permanent occlusion of a cerebral blood vessel (“*ischemic stroke*”) eventually leading to brain infarction. The final infarct size and the neurological outcome depend on a multitude of factors such as the duration and severity of ischemia, the existence of collateral systems and an adequate systemic blood pressure, etiology and localization of the infarct, but also on age, sex, comorbidities with the respective multimedication and genetic background. Thus, ischemic stroke is a highly complex and heterogeneous disorder. It is immediately obvious that experimental models of stroke can cover only individual specific aspects of this multifaceted disease. A basic understanding of the principal molecular pathways induced by ischemia-like conditions comes already from in vitro studies. One of the most frequently used in vivo models in stroke research is the endovascular suture or filament model in rodents with occlusion of the middle cerebral artery (MCA), which causes reproducible infarcts in the MCA territory. It does not require craniectomy and allows reperfusion by withdrawal of the occluding filament. Although promptly restored blood flow is far from the pathophysiology of spontaneous human stroke, it more closely mimics the therapeutic situation of mechanical thrombectomy which is expected to be increasingly applied to stroke patients. Direct transient or permanent occlusion of cerebral arteries represents an alternative approach but requires craniectomy. Application of endothelin-1, a potent vasoconstrictor, allows induction of transient focal ischemia in nearly any brain region and is frequently used to model lacunar stroke. Circumscribed and highly reproducible cortical lesions are characteristic of photothrombotic stroke where infarcts are induced by photoactivation of a systemically given dye through the intact skull. The major shortcoming of this model is near complete lack of a penumbra. The two models mimicking human stroke most closely are various embolic stroke models and spontaneous stroke models. Closeness to reality has its price and goes along with higher variability of infarct size and location as well as unpredictable stroke onset in spontaneous models versus unpredictable reperfusion in embolic clot models.

## Introduction

On average every 40 s someone in the USA suffers a stroke, which drastically demonstrates the omnipresence and frequency of this devastating disease [92]. Acute cerebral stroke is caused in the great majority of cases by occlusion of a supplying arterial vessel, whereas vessel rupture with associated hemorrhage accounts for a minority of about 15% [92]. The extent of the resulting brain injury after focal cerebral ischemia depends on many factors such as the severity and duration of ischemia or collateral blood flow to mention the most important. This is in contrast to global cerebral ischemia, which mostly occurs in the setting of cardiac arrest, where—under normothermic conditions—relatively well-defined survival times of various neuronal subpopulations, glial and endothelial cells exist [53]. For the sake of clarity, this review will focus on experimental models of ischemic stroke due to focal cerebral ischemia only. Because of its heterogeneous etiology with a broad spectrum of manifestations, a large repertoire of models is required to address specific facets of this complex disorder. The deep gap between ischemic stroke in experimental models and the reality of stroke in human patients becomes painfully illustrated by the fact that with the exception of recanalization of the occluded vessel none of the hundreds of experimental neuroprotective strategies could be translated into the clinic up to now [61]. Nevertheless, many aspects of the pathophysiology of focal brain ischemia have been primarily identified in experimental models and could be confirmed later also in the setting of human stroke [90]. The development of the concept of the penumbra, the identification of spreading cortical depolarizations, the detection of post-stroke neurogenesis and the phenomenon of preconditioning are prominent examples of fruitful experimental stroke research [25].

## Ischemic stroke in humans

What is the “reality” of ischemic stroke experimental models should depict? In humans, there are three different major causes for ischemic stroke. About 50% of cases are due to large vessel atherosclerosis and rupture of an atherosclerotic plaque, while about 20% are caused by cardioembolism. About 25% manifest as lacunar infarcts due to small vessel disease and probably occlusion of deep perforating arteries [9]. Some additional rare causes such as vasculitis or extracranial artery dissection account for the remaining 5% [129]. These percentages represent mean values over all age groups but change depending on the age of stroke victims. Cardioembolic stroke becomes the most frequent subtype with increasing age, while small vessel disease is rarely responsible in young people [121]. Furthermore, there are substantial differences in the distribution of stroke subtypes among different ethnic groups [113]. Finally, the percentages refer to ischemic stroke with known etiology, but one must keep in mind that a substantial number of cases are of undetermined cause, also referred to as cryptogenic stroke [114]. While atherosclerosis and cardioembolism frequently cause infarcts in both gray and white matter, lacunar infarcts are typically seen only in the subcortical white matter or the deep gray matter. Since these major subtypes of ischemic stroke already substantially differ concerning their RNA expression profiles in blood [62], it seems plausible that the molecular pathways and mechanisms of brain injury following ischemia may also be considerably different. In fact, this issue is currently unresolved. The neurological deficit and clinical presentation after ischemic stroke again exhibit a high variability with respect to the cause, duration, localization and severity of ischemia as well as age and comorbidity. One important fact in human thromboembolic stroke ignored in most available animal models is the occurrence of a substantial degree of spontaneous reperfusion, which is documented in up to 17% within the first hours after stroke onset [65]. However, reperfusion is mostly protracted but not prompt, thus leading to substantially different pathophysiologic pathways. While in the former situation the infarct core is irreversibly damaged and the penumbra is consumed within about 3 h, prompt reperfusion in animal models may partly rescue even core tissue with secondary delayed injury developing up to 3 weeks after stroke onset depending on the ischemic interval [55]. Peri-infarct blood flow may also be maintained by collaterals through the circle of Willis and/or leptomeningeal anastomoses. Clinically, stroke with acute onset of symptoms must be distinguished from transient ischemic attacks (TIAs) where neurological symptoms disappear within 24 h. In contrast to stroke, TIAs are thought to leave no damage of brain tissue, an opinion that may work conceptually but does not fully reflect reality [119]. In addition to acute neurological deficits that can be directly attributed to the affected brain areas, additional cognitive and psychiatric long-term consequences may emerge that are not readily assignable to the injured brain region. Cognitive decline is a major problem that is often present, in particular after lacunar stroke(s), and is probably superimposed to the underlying small vessel disease [85]. Post-stroke depression is another serious problem that develops in about one-third of patients with chronic stroke [105]. The biological basis remains uncertain, which is also due to a paucity of appropriate animal models [75]. Another example for frequent complications of chronic stroke widely ignored in animal models is obstructive sleep apnea [123]. Apart from neurological and neuropsychiatric consequences, stroke induces an immediate immune depression that is reflected by the fact that about 60% of stroke patients develop fever within 3 days after stroke onset [45]. A complex neuro-immunological connection consisting of the sympathetic nervous system, the hypothalamic-pituitary-adrenal axis and the vagus nerve has been identified as the biological basis [89].

Although stroke is a complex disorder, some common characteristics exist that can be modeled in experimental stroke. One important component of ischemic stroke is the evolving brain damage that is explained by the concept of the penumbra. Within minutes, reduction of the blood supply under 15–20% of baseline levels leads to an irreversibly damaged infarct core with rapidly evolving necrotic cell death. In the surrounding brain tissue blood flow is less reduced leading to loss of neuronal function while structural integrity is maintained. If there is no restoration of blood flow, this so-called tissue at risk will be incorporated into the infarct core. The range of cerebral perfusion between loss of electrical activity and irreversible neuronal depolarization has been termed penumbra [4]. This concept of the penumbra has become a milestone in stroke research since the penumbra can principally be rescued. Using perfusion-weighted and diffusion-weighted MRI methods, tissue at risk can be visualized by determining the perfusion-diffusion mismatch allowing an estimation of salvageable brain parenchyma [31].

Therefore, it is of outmost importance to be aware of the strengths and weaknesses of the many available experimental stroke models to choose the best one for the planned investigations where “best” means most closely mimicking a certain aspect of the multiple facets of ischemic stroke. One possible way to link findings in experimental stroke models to the reality of stroke in humans may be to add evidence by use of human brain tissue. For example, it should be tested whether findings in experimental animals concerning altered post-ischemic gene expression occur in a similar temporal, spatial and cellular pattern in human stroke. The same holds true for the verification at the protein level. Currently, excellent experimental stroke papers only sometimes include such investigations using tissue from one or at best a few autopsied human brains. Although this represents a first and important step, the quality criteria for experimental stroke such as blinding, randomization, definition of inclusion/exclusion criteria and a reasonable sample size calculation [66] should also be applied to the analysis of human autopsy cases. Failure of successful translation of experimental strategies into patients may also be caused by lack of precise information about whether the therapeutic target is present and regulated similarly in human stroke. In this context, the urgent need to build up large stroke brain banks becomes apparent, an issue that is ignored in many countries.

## General issues concerning models of ischemic stroke

### Genetic and epigenetic differences

There is a series of general issues independent from the choice of model potentially conflicting the reality of human stroke in the experimental setting. Although it may appear trivial, awareness of the genetic and epigenetic differences between mice and rats (the most frequently used animals in stroke research) and humans did not increase until the last few years. There is growing evidence that this issue indeed may have been given too little attention when translating results from animal experiments to human patients. Comparing the expression profiles by deep RNA sequencing of 15 tissues from mice and humans including brain, the expression for many sets of genes was found to be more similar in different tissues within the same species than between the two species [81]. Associated epigenetic histone marker analysis further corroborated these findings. Another example for substantial differences between murine and human epigenetic regulation is the *NOS2* gene in macrophages. In contrast to mice, the human *NOS2* gene is silenced by CpG methylation, suggesting that there may be yet widely neglected differences between human and murine macrophages concerning their reaction to inflammatory stimuli [44]. In a most recently published proof-of-concept study, it could be shown that both baseline expression of chemokines/cytokines and the response after oxygen-glucose deprivation in primary neurons, astrocytes and microglia differed significantly between rodents and humans [29]. It is reasonable to assume that these species differences may have an impact on stroke pathophysiology. This is further corroborated by results from a multiplex immunoassay characterization of chemokine/cytokine response demonstrating significant differences after experimental stroke in mice compared to the analysis of autopsy material from patients suffering from ischemic stroke [96]. Last but not least, the gut microbiome and its influence on the immune response is attracting growing attention. Although this issue may be overinterpreted currently [133] considering that there are already significant differences in the fecal microbiota of inbred strains from different vendors [34], the impact on the modification and variability of experimentally induced diseases including stroke may be substantial.

### Differences in brain anatomy and functional organization

**Fig. 1:**
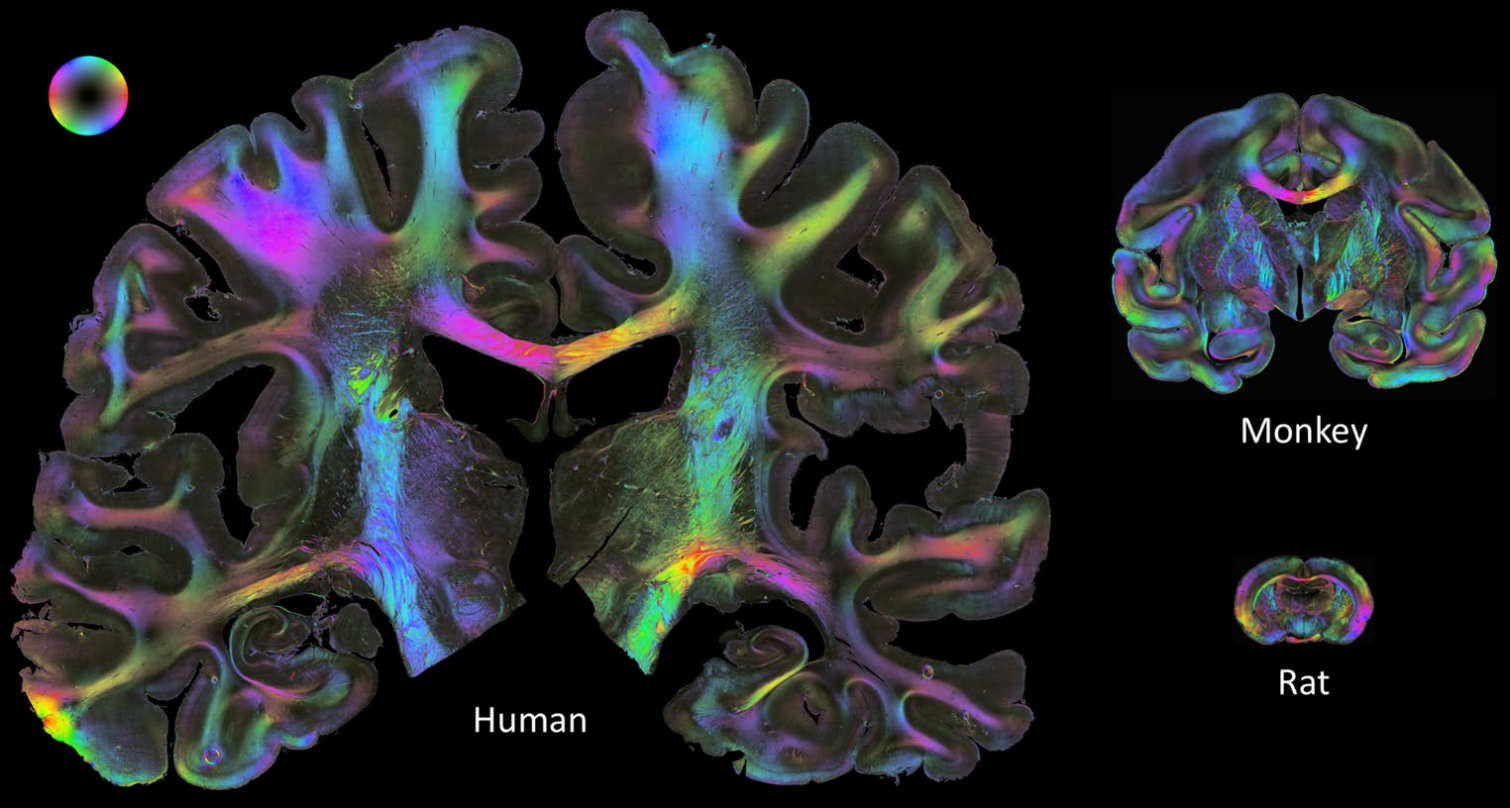
Illustration of connectivity as obtained by 3D-polarized light imaging (3D-PLI) [5, 6, 139]. Coronal sections from a human brain, a vervet (monkey) and a rat brain. The *color sphere* indicates the direction of fibers; *black* corresponds to a steep course of fibers in the third dimension, within the physical section. The comparison illustrates that not only the absolute volume of brains differs among the three species, but also the relative amount with a much higher proportion of white matter in human brains as compared to vervet and rodent brains. (Images by courtesy of Markus Axer, Karl Zilles and Katrin Amunts, Forschungszentrum Jülich, Germany)

The macroscopic differences in brain anatomy between humans and other species are evident at first sight. With few exceptions, in vivo stroke models use animals with a lissencephalic brain. However, whether this difference is a major drawback for stroke models is under debate [20]. Nevertheless, the different anatomical and functional organization becomes relevant with respect to the infarct localization. While in animal models the main focus is frequently on infarct size, this reflects reality in human conditions only to a limited extent. The localization of the ischemic lesion within particular connections may be more relevant for the clinical syndrome and long-term outcome (Fig. 1). Another serious issue is the low extent of white matter in rodents compared to humans. While in humans the percentage of white matter accounts for 60%, it decreases to about 35% in dogs, 20% in rabbits, 15% in rats and is as low as 10% in mice [73] (Fig. 1). However, ischemic damage of the white matter plays an important role for the prognosis of stroke outcome and is the major cause of hemiparesis in all three major stroke subtypes [1]. In a very recent study the relevance of remote white matter integrity for long-term cognitive outcome has been clearly demonstrated [110]. In lacunar stroke, white matter damage even plays the central role. Furthermore, while the differences in the complexity and functional organization of the brain are less problematic when analyzing the molecular and cellular responses after acute ischemic stroke, it becomes a major issue in models of chronic stroke where recovery of function and neuropsychiatric long-term consequences are of particular interest. Interestingly, although pronounced lateralization of brain function is one feature of human brains, it is also already present in rodents. While rats subjected to right MCA occlusion exhibited transient hyperactivity, this effect was not seen after occlusion of the left MCA [104].

### Differences in vascular anatomy

To protect the human brain from fluctuations of blood supply, several safeguards have merged during evolution. First, redundancy of cerebral perfusion is provided by three collateral systems in the cerebral vasculature. One principal shunt exists between the branches of the extra- and intracranial arteries. Furthermore, the circle of Willis at the base of the brain with connections between the anterior and posterior circulation as well as between the left and the right hemisphere allows redirection of the blood flow when one artery becomes occluded. However, a high interindividual variety of the circle of Willis has been shown in many studies with a completely normal configuration only in a minority of cases without evidence for ethnic differences [32]. The most complex collateral system is represented by a highly interconnected dense network of leptomeningeal vessels protecting in particular cortical structures unless blood pressure is high enough [12]. Second, at a functional level perfusion can be regulated by autoregulation of cerebral resistance arteries and endothelial regulation of the vascular tone by releasing vasoconstrictive or vasorelactant molecules. Neuronal activity is also closely linked to enhanced perfusion a phenomenon termed neurovascular coupling [91]. It is immediately apparent that alterations and even variations in these systems will influence survival of brain tissue after focal ischemia, and it is also obvious that differences in these systems between species will result in different patterns of ischemic damage.

**Fig. 2:**
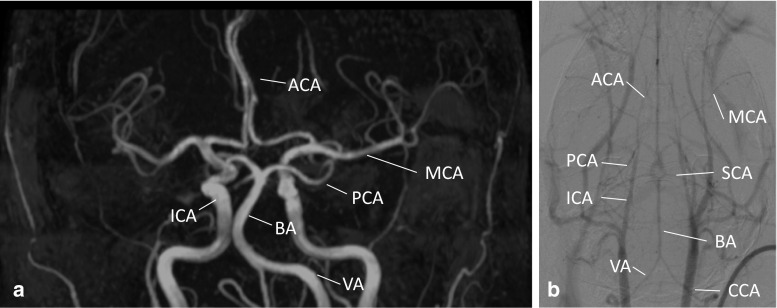
Imaging of the human (**a**) and murine cerebrovascular system (**b**). Although the principle plan of cerebrovascular organization is similar in humans (**a**) and mice (**b**), some differences exist. The BA, for example, in humans most often terminates by splitting into the PCA, whereas in mice the BA in most cases terminates by splitting into the SCA (with the PCA originating from the ICA). Also, in humans the postcommunicating ACAs normally are paired, whereas mice feature an azygos ACA. **a** Time-of-flight MR angiography showing the human arterial cerebrovascular system, (maximum intensity projection, AP view); **b** in vivo digital subtraction angiography (DSA) of the murine cerebrovascular system [35, 111]. Images courtesy of Prof. Marc A. Brockmann, Department of Neuroradiology, University Medical Center of the Johannes Gutenberg University, Mainz, Germany. *ACA* anterior cerebral artery, *BA* basilar artery, *ICA* internal carotid artery, *MCA* middle cerebral artery, *PCA* posterior cerebral artery, *SCA* superior cerebellar artery, *VA* vertebral artery

Although the principle plan of vascular organization is present in all mammalian brains, there is a broad spectrum of variations between species and even strains concerning the number and diameter of collaterals in arterial trees and the capacity for remodeling after vessel occlusion (Fig. 2). In C57Bl/6J mice, for example, a complete circle of Willis is present in only 10% [88]. Compared to BALB/c mice, substantial differences are present in the total collateral system with candidate genes predominantly on chromosome 7 identified in association mapping studies and suggesting a high polymorphism [127]. Similarly, a genetic background could recently be related to variations in the circle of Willis of gerbils [80]. Wistar rats have been shown to possess thinner posterior communicating arteries than Sprague-Dawley rats [68]. Sprague-Dawley rats on the other hand have atypical branching of the MCA in nearly 20% [38]. This partly explains the high variance of infarct sizes even in the same species or strain after occlusion of an artery. Another aspect is that the variability of the vascular anatomy impedes specific models of artery occlusion. Due to a network of freely anatomizing arteries, the so-called “rete mirabile,” a proximal occlusion of the MCA for infarct induction, is not suitable in larger animals such as cats, dogs, sheep, goats and pigs [56]. In contrast, collateral blood flow in rodents after MCA occlusion is generally poor [54].

While differences in the gross anatomy of arteries and collaterals may cause only variations in brain injury patterns, functional differences may have deeper implications concerning the pathophysiology of the ischemic cascade. At least for neurovascular coupling after ischemic stroke differences between humans and animals have been described with generally impaired functional hyperemia in animals but variable findings in stroke patients [60]. Another structure playing a key role in the pathophysiology of stroke is the blood-brain barrier (BBB). Vessel occlusion with breakdown of the supply with oxygen and glucose damages not only neurons and glial cells resulting in cytotoxic brain edema, but also injures the BBB itself additionally causing a vasogenic brain edema. There is some evidence that ischemic damage of the BBB in humans may primarily involve other components of the vascular tree. While in both mice and rats albumin extravasation could be identified around arteries, capillaries and veins in human autopsy tissue, the capillary compartment was relatively spared [76].

### Differences in the immune system

While in the last decade much interest focused on the inflammatory response after stroke, the differences between rodents and the real situation in humans are immense. This starts with the simple fact that the percentage of neutrophils in mice and rats is about 10–20% compared to 50–70% in humans, while the opposite situation is seen for lymphocytes, which comprise about 50–100% in rodents compared to 20–40% in humans, respectively [47]. Moreover, there is only a minimal intersection of whole-genome mRNA and microRNA expression in leukocytes from rodents versus humans at both baseline and after stroke, raising the question whether rodents are acceptable models at all for the human immune system after stroke [116]. The subnetworks of chemokines/cytokines in human, mouse and rat brain cells differ after in vitro ischemia [29], and there are important differences between microglia from rodents and humans [118]. How minimal differences in the immune system even between—compared to rodents—relatively closely related species, namely the gyrencephalic cynomolgus macaque and humans, may cause substantially different responses became dramatically apparent in the TGN1412 trial. In 2006, six healthy volunteers suffered from a life-threatening incident by developing a cytokine-release syndrome with multiple organ failure in a phase I clinical trial after administration of the CD28 super agonist antibody TGN1412, an immunomodulatory drug. In contrast to CD4+, T cells of humans expressing CD28 macaques lost CD28 expression of CD4+ T cells during differentiation [58]. This detail has been overlooked in preclinical testing and resulted in the different immune responses although the dose in monkeys was up to 500 times higher. Since inflammation after ischemic stroke is thought to play a crucial role in the pathophysiology of the ischemic cascade and also with regard to the final outcome, one should always be aware of the potential fundamental differences between animal models and reality.

### Characteristics of experimental animals

Perhaps the most obvious discrepancy between reality and experimental stroke models is the difference in the populations analyzed. Ischemic stroke in humans preferentially occurs in elderly patients of both sexes with often multiple comorbidities (diabetes mellitus, hypertension, hyperlipidemia, obesity) requiring multiple medications with multiple complex interactions. In contrast, the vast majority of experimental stroke studies have been performed on young, healthy, male inbred rodents housed under optimal pathogen-free conditions [24]. Experiments are performed under highly standardized and very well-controlled conditions to reduce the variability of infarct size to a minimum. In a recent review on strain-related differences in the immune response and the relevance to human stroke, Becker [11] provocatively but concisely formulated: “… almost all of what we know about the role of the immune system in stroke is derived from a single line of inbred mice.”

### Anesthesia

Another basic difference between experimental models and the reality of stroke occurring in humans is the use of anesthesia in the majority of animal models. Apart from peripheral effects, including the influence on blood pressure, cerebral blood flow and metabolism, anesthetics may have neuroprotective effects, thus modulating some aspects of spontaneously occurring post-ischemic processes [69, 71].

## In vitro models of ischemic stroke

It is clear that the complex situation of ischemic stroke cannot be modeled in an in vitro system with single cells or pieces of brain tissue with the absence of intact blood vessels and blood flow as well as the lack of infiltration of leukocytes. Nevertheless, in vitro models allow the investigation of specific basic biochemical and molecular mechanisms under conditions of energy deficiency similar to ischemia. The fundamental critical control points and molecular pathways of necrotic cell death, programmed cell death and autophagy are also amenable to direct study in vitro [52]. Another advantage of in vitro models is the possibility of high-throughput analyses, which becomes relevant with respect to testing novel potentially neuroprotective pharmaceuticals. In this context, the possibility to use human or humanized cells becomes increasingly important.

There are two principal ways to induce ischemia-like events outside a living organism: either the deprivation of oxygen and glucose or the chemical or enzymatic blockade of the cellular metabolism. The most frequently used in vitro model of cerebral ischemia is the combined oxygen and glucose deprivation (OGD). To this end, the atmosphere in the incubator with cultured cells or brain slices is exchanged. The normal O_2_/CO_2_ equilibrated medium is replaced by an N_2_/CO_2_ equilibrated medium in a hypoxic chamber with glucose omitted from the medium. Retaining glucose in the hypoxic chamber is termed hypoxia and is less suitable for modeling an ischemic event, which always is accompanied by breakdown of the nutrient supply. Compared to in vivo models, there is characteristically a need for a longer episode of energy deficiency to induce neuronal death. Typically, cell cultures are exposed to OGD for 1–24 h. In contrast to single-deprivation paradigms, 1 h exposure to OGD is already sufficient to induce widespread neuronal death [42]. Modeling ischemia-reperfusion can be achieved by a return to standard culture conditions. A particular aspect of the ischemic cascade is excitotoxicity, resulting from an increase in glutamate with consecutive overactivation of glutamate receptors. In vitro models allow for an isolated analysis of this specific component of the ischemic cascade by exposing the culture to glutamate or other specific agonists of excitatory receptors [19].

A broad repertory of cellular systems to model in vitro ischemia exists, excellently reviewed by Holloway and Gavins [52]. The two main cellular platforms, organotypic brain slices and primary cell cultures, respectively, will briefly be introduced. The organotypic brain slice has the advantage of a functional system with preservation of the neuronal morphology and the presence of glial cells and network connections in particular when using hippocampal slices. On the one hand, lack of perfused vessels in brain slices clearly represents an artificial situation that on the other hand allows separating the ischemic effects on neuronal tissue from those due to actions on the cerebrovascular system [28]. Since all in vitro systems primarily mimic the situation of global cerebral ischemia, there are attempts to more closely model the situation of ischemic stroke, i.e., focal cerebral ischemia [101]. To this end, OGD medium is only focally applied to the brain slice while the rest is kept in normal oxygenated media. Interestingly, neurons adjacent to the OGD core progressively depolarize, a phenomenon seen in the penumbra in vivo. Thus, the situation comes closer to the situation of focal cerebral ischemia. Most importantly, brain slices can also be prepared from human brain tissue and exposed to OGD experiments [102]. To study the cell-specific responses to stress such as OGD, the use of primary neuronal and glial cells is an invaluable benefit to understand their specific roles in stroke pathophysiology. These studies represent an important component of preclinical stroke research but have to be combined with in vivo experiments to come closer to the reality of human stroke. Modeling the BBB is another important in vitro approach to understand one structure that plays a key role in stroke pathophysiology. Since it has become clear that the BBB is only one component of the neurovascular unit consisting of endothelial cells, pericytes, astrocytes, oligodendrocytes, microglia and neurons, i.e., practically any cell type in the brain [22], most recent approaches try to model this complex system with all components three-dimensionally in vitro [3].

## Rodent models of ischemic stroke

While in the beginning of experimental stroke research mainly higher species were used, this situation has completely changed within the last 3 to 4 decades. Today, mostly mice and rats are chosen for in vivo stroke models, which is easy to understand considering the lower costs of acquisition and keeping, simpler monitoring methods and tissue processing as well as ethical issues. The possibility to easily create transgenic animals is another major advantage of mouse models. However, one always has to keep in mind that the results of rodent models of stroke may only be half the truth. Due to the constant failure to translate experimental neuroprotective therapies into patients, the “stroke community” stands in the forefront of critically scrutinizing to what extent experimental models reflect reality. Starting with the STAIR recommendations 1999 [122], a series of landmark reviews considering this issue and providing recommendations to model stroke more realistically in the experimental setting has been published [26, 33, 36, 84]. In particular, there is the strong advice to reproduce successful stroke therapy in rodents in a higher species before starting clinical trials. The following paragraphs will briefly discuss the most important models with a focus on differences and similarities to the situations in humans (Table 1). For an extensive and excellent overview of currently available models with a focus on methodological aspects, see [23].

**Table 1:** Ischemic stroke: experimental models and reality

Experimental model	Close to reality	Far from reality/distorting reality
In vitro models	Principal mechanisms and molecular pathways of cell death under ischemia-like conditions	Absence of intact blood vessels and blood flow Lack of infiltration of leukocytes
Endovascular suture model	Localization of the infarct (mostly MCAO), penumbra, blood-brain barrier injury, inflammatory processes and cell death pathways (Permanent and transient ischemia) No craniectomy	Large infarcts, mimics rather malignant infarction [16] Involvement of the hypothalamus with consecutive hyperthermia (rat) [79] Prompt reperfusion by withdrawal of the filament [55] Exception: mimics closely endovascular mechanical thrombectomy Thromboembolism/thrombolysis not modeled Anesthesia
Craniectomy models with direct vessel occlusion	Penumbra, blood-brain barrier injury, inflammatory processes and cell death pathways (Permanent and transient ischemia)	Prompt reperfusion by reversal of the mechanical occlusion [55] Exception: mimics closely endovascular mechanical thrombectomy Thrombembolism/thrombolysis not modeled Craniectomy Anesthesia
Photothrombotic stroke	Small cortical infarcts and small subcortical infarcts (Permanent ischemia only) Recovery and plasticity mechanisms in chronic stroke Modifications with stroke induction in freely moving rats and mice allow real-time analysis of a number of parameters in acute stroke without distortion through anesthesia [81, 136]	Simultaneous development of cytotoxic and vascular edema with rapid breakdown of the blood-brain barrier No penumbra (whether the “ring” model accurately models penumbra under discussion) [16] Anesthesia
Endothelin-1 model	Infarcts of variable sizes in nearly any brain region Subcortical stroke Recovery and plasticity mechanisms in chronic stroke (Transient ischemia only)	Minimal edema [112] Endothelin-1 and endothelin-1 receptors present also on neurons and astrocytes [94, 95]—may interfere with post-stroke recovery mechanisms [16]
Thromboembolic clot models	Thromboembolic infarcts Transient ischemia with unpredictable time point of lysis of the embolus Pathophysiology of embolic stroke including primarily cytotoxic edema superimposed later on by vasogenic edema with breakdown of the blood-brain barrier, presence of a penumbra, development of spreading depressions as well as an inflammatory response Possibility to test thrombolytic therapies	(Animal model)
Microsphere models of embolic stroke	Thromboembolic infarcts (Permanent ischemia) (Mini-)penumbras, pathophysiology of ischemic cell death, inflammation	Permanent ischemia without possibility of reperfusion Multiple vessels occluded Capillaries and arterioles are blocked resulting in redistribution of the blood flow and immediate disruption of the blood-brain barrier and vasogenic edema [132]
Macrosphere models of embolic stroke	Thromboembolic infarcts (Permanent ischemia) Pathophysiology including penumbra, ischemic cell death, inflammation Occlusion can be postponed allowing to induce ischemia while the rat lies in an MRI or PET scanner	Permanent ischemia without possibility of reperfusion
Spontaneous stroke models: spSHR rat	Subcortical infarcts (Small) vessel pathology	(Animal model)

### Intraluminal suture MCAO model

**Fig. 3:**
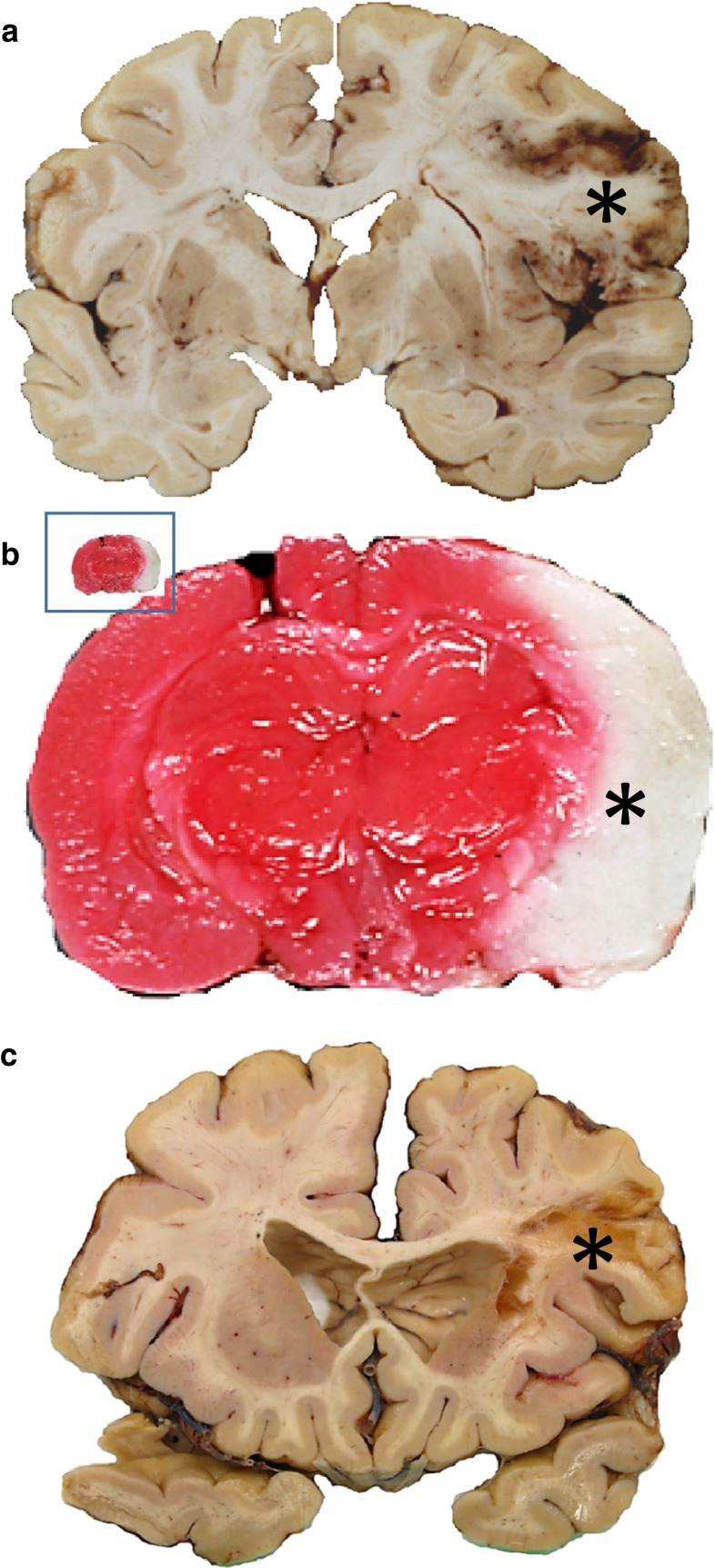
Media infarcts in human (**a**, **c**) and rat brain (**b**). An acute ischemic stroke in the territory of the middle cerebral artery (MCA) in a human brain (**a**; *asterisk* infarct). Such acute large media infarcts are easily modeled with the filament technique (**b**; rat, 120 min transient ischemia, 24 h survival; TTC stain: vital tissue red, white tissue (*asterisk*) indicates infarct; inset: real proportions of the rat brain compared to human brain. Chronic infarcts (**c**, human) of similar sizes in rodents rarely come to examination

The most frequently used experimental model of ischemic stroke in rodents is the intraluminal suture middle cerebral artery occlusion (MCAO) model, which does not require craniectomy. To induce vessel occlusion, a monofilament is introduced into the internal carotid artery and advanced until the origin of the MCA is blocked. This method was developed and first described by Koizumi and colleagues [72] in rats followed by multiple modifications particularly concerning the kind, coating and length of the filament as well as modifications of the access route (e.g., [82]). Meanwhile, the model has also been adopted for mice and is increasingly applied there [49]. Principally, the suture model can be used to model permanent ischemia or by withdrawal of the filament with subsequent reperfusion as a model for transient focal cerebral ischemia with variable reperfusion time points. Typically, reperfusion intervals range between 60 and 120 min in rats, resulting in delayed neuronal death or pannecrosis of large parts of the ipsilateral hemisphere, respectively. Since most thromboembolic infarcts in humans occur in the territory of the middle cerebral artery (MCA) [98], this model closely resembles manifestation of human stroke concerning the localization (Fig. 3). Inherent to this model is involvement of the hypothalamus after occlusion times of 120 min or longer in rats resulting in spontaneous hyperthermia, which is rarely seen in humans [41, 79]. Concerning the infarct sizes, Carmichael pointed out that the suture MCAO method in rodents may frequently model malignant infarction with progressive edema rather than most cases in humans with comparatively small infarcts [16]. Although the intraluminal suture model has provided substantial knowledge of the pathophysiology of cerebral ischemia in particular concerning the ischemic penumbra, blood-brain barrier injury, inflammatory processes and cell death pathways, it has a serious inherent problem. In human ischemic stroke, vessel occlusion is frequently not complete, and at least partial spontaneous reperfusion due to successive resolution of the thrombus occurs in most patients in the first 48 h after stroke [65, 137]. In contrast, withdrawal of the filament in the suture model results in an ad hoc reperfusion. This entails induction of molecular and cellular mechanisms more similar to the situation after global ischemia but is entirely different compared to the human situation. Although it is currently the most frequent model of ischemic stroke, on a somewhat more critical note, one can say that it does not fit any of the three major subgroups of stroke with regard to the etiology. In a provocative opinion article, it was suggested to take this model completely out of the repertoire of stroke models because its clinical relevance in particular with respect to translational aspects is poor [55]. Even rtPA-induced lysis of a thromboembolic occlusion is associated with prolonged reperfusion deficits. However, the suture model may closely mimic the situation of interventional mechanical thrombectomy. In 2015, five multicenter randomized clinical trials were published demonstrating beneficial effects of endovascular therapy of ischemic stroke due to large vessel occlusion [43]. In light of these results, increasing clinical use is to be expected making the filament model indeed more relevant for this specific condition.

### Craniectomy models

Another principal possibility for induction of focal ischemia is to directly occlude a cerebral vessel. If this is done by clipping, ligation or with hooks, reperfusion is principally possible. Cauterization followed by transection on the other hand results in permanent ischemia. All these direct approaches have in common that a craniectomy with incision of the dura mater is necessary. This distorts the reality of human stroke in so far as craniectomy represents a kind of skull trauma that may induce cortical spreading depressions and inflammation. In unfavorable cases, the brain itself may be directly injured by the drill or thermally damaged by electrocoagulation of a vessel. Due to the opening of the dura, the brain is exposed to the atmosphere, the intracranial pressure is affected, and changes in regional brain temperature may occur [23]. The first description of MCA occlusion via a direct approach was made by Robinson et al. [106] in Sprague-Dawley rats using ligation of the distal MCA to induce cortical ischemia. Tamura developed a model with a more proximal occlusion of the MCA resulting in infarcts in the cortex and striatum similar to the suture model but without the hyperthermic response [125]. Two of the most frequently used modifications are the tandem vessel occlusion with electrocoagulation of the distal MCA plus unilateral common carotid artery (CCA) occlusion [14] or the so-called three-vessel occlusion model with additional bilateral CCA occlusion [18] resulting in large cortical infarcts with high mortality. These craniectomy models with their multiple variations are used in many species including non-human primates. One has to keep in mind that prompt reperfusion by reversible mechanical occlusion comes up with the same problem discussed for the suture model. The pathophysiological consequences are far from the reality of ischemic stroke in humans with spontaneous or rtPA-induced opening of the occluded vessel [55].

Models of ischemic stroke in the posterior cerebral circulation to mimic vertebro-basilar stroke are technically elaborated and are mostly performed in large animals. A vertebro-basilar stroke model in the rat has been described by Henninger et al. [50].

### Photothrombosis model

The photothrombotic stroke model (synonymously also named the photochemical stroke model) has been primarily developed in rats [130], but meanwhile modified protocols for mice are available [67]. For stroke induction, a photosensitive dye (e.g., Rose Bengal) is systemically applied followed by illumination of the brain through the intact skull with light of a specific wavelength. The illumination activates the dye leading to the formation of singlet oxygen and superoxide, which in turn results in endothelial injury, platelet activation and aggregation. This causes rapidly evolving ischemic cell death in the illuminated brain area in end arterial regions eventually leading to a cortical ischemic lesion. Whether platelet activation really plays a major role is a matter of debate, so “photothrombotic” model may indeed be a misnomer [70]. One advantage of this model is the possibility to select a specific cortical brain region of interest for the ischemic lesion because of the use of stereotactic coordinates and application of the activating light at the desired area. Another advantage is its high reproducibility or the other way around the minimal variation in infarct size combined with very low mortality. This makes it a predestined model to study repair mechanisms and related long-term functional outcome [21, 39]. The major issues with this experimental model are fundamental discrepancies compared to the situation in acute human stroke. While the latter is characterized by a primarily cytotoxic edema [109], photothrombotic stroke induces a nearly simultaneous development of cytotoxic and vascular edema with rapid breakdown of the blood-brain barrier. The second major drawback is a virtual lack of a penumbra and collateral blood flow. Since the penumbra or the tissue at risk is the major target of any neuroprotective therapy, the translational impact of this model is poor. Modifications of the classical model have been developed to mimic also a penumbral region. Adapting the illumination parameters by use of a ring filter (the so-called “ring model”) results in a central area without thrombosis surrounded by damaged brain [131], but whether this adequately corresponds to the penumbra in the human situation is under discussion [16]. Other modifications include the targeting of individual brain arterioles or the induction of small subcortical infarcts by implanted optical fibers to model lacunar stroke. Another interesting approach is the induction of photothrombotic stroke in freely moving rats and mice, which allows real-time analysis of a number of parameters in acute stroke without distortion by anesthesia [83, 136].

### Endothelin-1 model

Endothelin-1 is a peptide with potent and long-lasting vasoconstrictive properties [135]. Application onto an exposed vessel or directly on the brain surface or stereotactically injected into the brain parenchyma leads to vasoconstriction inducing downstream ischemia [37]. Using this method, more or less any region in the brain is targetable. Depending on the concentration of endothelin-1, the severity and duration of ischemia as well as the resulting infarct size may be modified. When the endothelin-1 effect eases, blood flow is gradually reestablished, thus representing the situation of transient focal ischemia. The model has been developed in rats [103] and modified for the use in mice [107, 120]. Although endothelin is considered to play a key role in the induction of vasospasms after subarachnoid hemorrhage with subsequent infarcts in human patients, there are no data on its application in animal experiments to model this specific situation. Since ischemia develops slowly after endothelin-1 application and is accompanied only by minimal edema, this model again does not accurately mimic human stroke [112]. Similar to the photothrombotic model, it may be more useful for simulating lacunar stroke and for long-term studies with a focus on recovery mechanisms. However, endothelin-1, endothelin-1-converting enzyme and endothelin-1 receptors are not only expressed by endothelial cells, but are also present on neurons and astrocytes [94, 95]. Even more, endothelin-1 application has been shown to induce proliferation of astroglia, producing a permissive milieu for enhanced axonal sprouting, which may interfere with post-stroke recovery mechanisms [16].

### Embolic stroke models: thromboembolic clot models

Embolic stroke models fall in two major categories: thromboembolic clot models and non-thromboembolic, microsphere or macrosphere-induced stroke models. All the variants of embolic stroke models go back to the first description of a thromboembolic infarct model in rats by Kudo et al. [77]. Apart from the general issues of animal models mentioned above, the clinical reality of human embolic stroke is in fact best modeled using any kind of embolus-like material that is introduced into the cerebral vessel system, typically in the extracranial internal carotid artery (ICA) or a stump of the external carotid artery (ECA). In thromboemblic models, the clot can be gained from spontaneously formed or thrombin-induced thrombotic material, mostly from autologous, but also from heterologous blood [30]. Depending on the size and number of clots as well as the application route, this will cause occlusion of one or multiple vessels followed by infarcts in the respective supplied territory. Alternatively, thrombin can be directly injected into the ICA or MCA to mimic vascular occlusion. However, the composition of the clot differs substantially from that in humans since it consists primarily of fibrin with only few cells that may influence spontaneous or therapeutically induced lysis [99].

Similar to the situation in humans, there is a high degree of variation in the infarct localization and infarct size. More importantly, there is unpredictable partial or complete lysis of the occluding material followed by reperfusion, which resembles the clinical situation more closely compared to all the other experimental situations. Thromboembolic clot models are therefore ideal for analysis of spontaneous or iatrogenic lysis by rt-PA. The pathophysiology of embolic stroke in animals perfectly matches human embolic stroke including primarily cytotoxic edema superimposed later on by vasogenic edema with breakdown of the blood-brain barrier, presence of a penumbra, development of spreading depressions as well as an inflammatory response [54]. Not only the acute but also the chronic phase with spontaneous recovery mechanisms can be analyzed. This advantage of great similarity goes along with the disadvantage of great variability and consequently the need for higher numbers of animals to achieve statistically meaningful results. This led to the development of embolic material not lysable for better standardization but at the price of departing from reality.

### Embolic stroke models: microsphere/macrosphere stroke models

Many different materials such as silicone, collagen or titanium dioxide (TiO_2_) have been used for embolic stroke induction in animal models [30]. The basic difference compared to the thromboembolic clot model is that the artificial spheres do not dissolve and therefore cause permanent ischemia. Microspheres have a diameter of 20–50 µm and cause microembolization of multiple vessels resulting in multifocal and heterogeneous infarcts that develop up to 24 h after injection [87]. Primarily developed to mimic transient ischemic attacks, it can also be used to induce graded infarcts depending on the size and number of emboli [54]. The pathophysiology, however, differs from the typical human condition of embolic stroke with occlusion of one artery. In the microsphere model, capillaries and arterioles are blocked resulting in redistribution of the blood flow and immediate disruption of the blood-brain barrier and vasogenic edema [132].

Macrospheres have diameters between 300 and 400 µm, which are installed in the ICA and result in well-defined infarcts in the MCA territory [40]. Again, the severity of the ischemia and infarct size can be shaped by the number of up to six macrospheres. Kinetics of infarct development, lesion site and infarct size as well as outcome are similar to the intraluminal suture model. However, the hypothalamic artery is not blocked, and, compared to the intraluminal suture model, hyperthermia due to hypothalamic injury does not develop. An advantage of this model is that the occlusion can be postponed, allowing to induce ischemia while the rat lies in an MRI or PET scanner (review by [126]).

## Modeling risk factors and comorbidity

In particular, the persistent failure to translate successful neuroprotective strategies in rodents or even non-human primates into human patients suffering from ischemic stroke led to critical reflections about whether the animal models used are indeed suitable to cover the reality of human stroke [33]. Apart from possible experimental quality problems, the use of young, healthy, male rodents in the overwhelming number of experiments is a major distorting factor. Therefore, in the last years numerous attempts have been made to use animal models with the most frequent comorbidities such as diabetes mellitus, hypertension, atherosclerosis, hyperlipidemia, obesity or infection for stroke research. Further, aged animals and animals of both sexes have been used to capture reality more accurately. Age is the single most unmodifiable risk factor for stroke. From experimental studies with aged animals, it has become clear that neurological impairment increases, whereas the regenerative capacity is lowered compared to younger animals (for review, see [15]). There is growing evidence that the common link of age and the various modifiable risk factors mentioned above may be an elevated inflammatory profile resulting in a stroke-prone state [93, 108]. Sex is another important factor significantly affecting stroke incidence and outcome [51].

When modeling risk factors of stroke, two principal experimental strategies are available. The most realistic approach would be to develop animal models with inherent risk factors that eventually result in spontaneous stroke. This approach is widely restricted to animal models of hypertension. Although closely mimicking the situation of human stroke, these models are costly and extremely time-consuming. The other principal option is to induce ischemic stroke in animals (or stroke-like conditions in in vitro systems) with preexisting comorbidities. The stroke models per se are principally the same as described in the above paragraphs. The major challenge in designing experimental ischemic stroke is to find a good balance between the high complexity of multiple interacting parameters as in humans and pragmatically manageable studies delivering good results for specific scientific problems.

### Spontaneous stroke models

Since hypertension is the single most important modifiable risk factor for stroke in humans [2], the use of hypertensive animals for stroke research is a step in the right direction to create a more accurate situation of clinical reality. The models most frequently used are the spontaneously hypertensive rat (SHR) and the stroke-prone spontaneously hypertensive rat (spSHR), which were developed about 50 years ago by Okamoto and Aoki [97]. The SHR is a selective inbreed from Wistar-Koyoto rats that developed the highest blood pressure values, while the spSHR is one substrain of the SHR. This phenotype selection, however, precludes appropriate controls matched for both hypertension and genetic background. The SHR is normotensive at birth and develops hypertension because of an overactivity of the renin-angiotensin system between 2 and 4 months settling at systolic values of about 200 mmHg by 6 months. While the SHR rarely suffers from spontaneous stroke, the spSHR develops hypertension under a high-salt diet from the age of 6 weeks, which rapidly aggravates to malignant hypertension (>240 mmHg) by 12 weeks accompanied by cortical strokes and hemorrhages in the frontal, parietal and occipital regions by about 20 weeks [8]. The spSHR also develops small subcortical infarcts in the striatum and subcortical white matter because of hypertensive small vessel disease with arteriolar wall thickening, fibrinoid necrosis and enlarged perivascular spaces similar to the human pathology [7]. Pathophysiologically, the first step seems to be an alteration of the endothelial tight junctions that occurs before hypertension and hypertension-related vessel changes or even ischemic lesions develop, a mechanism quite controversially discussed for human small vessel disease [8, 10, 13, 48, 128]. A certain degree of caution with the translation of the findings in spSHR is also indicated when considering that due to the unpredictable onset of stroke the discrimination between causative and reactive changes may be not always clear. Also noteworthy is the fact that there are already substantial differences in vessel structure between spSHR and SHR compared to their Wistar-Koyoto parent strain. The media-to-lumen ratio in the cerebral arteries is increased in SHR and spSHR compared to Wistar-Koyoto rats, which may be a risk factor under reduced cerebral blood flow with significant changes in pathophysiology and outcome [64]. Nevertheless, the spSHR is currently by far the best animal model for lacunar stroke in humans because of the similarity of the underlying small vessel disease [46]. For the sake of completeness, other spontaneous stroke models such as the male-inducible hypertensive rat or (R+/A+) mice, which are double transgenic for the human renin and human angiotensinogen genes, are available, which also develop spontaneous ischemic and hemorrhagic cerebral lesions after induction with specific diets [46].

## Special comment on non-human primate models

Non-human primates may have a lissencephalic brain, like the common marmoset and the squirrel monkey, or they may possess gyrencephalic brains, like the baboon, the Rhesus macaque and the Cynomolgus macaque, to mention the most relevant species in the context of stroke models. Of all primates, the macaque monkeys most closely resemble the human brain concerning anatomy with a specific cortical and subcortical organization, but most important for stroke research also with regard to vascular supply and collateralization [20]. Nevertheless, currently it is unclear which non-human primates most closely mimic the human conditions of stroke.

For a long time, above all the baboon model has been used in stroke research. Although these non-human primates have a gyrencephalic brain, the rich collateralization compared to humans demands relatively complex techniques for stroke induction with many modifications available [78]. Typically, a transorbital route with enucleation of the eye has to be performed to get access to the MCA and both anterior cerebral arteries to induce infarcts in the cortex and subcortical white matter [57]. This, however, prevents post-stroke behavioral tests requiring binocular vision. Depending on the stroke syndrome to be mimicked, all the various models of ischemia induction are principally possible including endovascular models with balloon occlusion [134] similar to the suture model in rodents.

Although one would intuitively postulate that experimental stroke in non-human primates is closer to the situation in humans, there are some data that may make one thoughtful. Apart from one single study, there is a lack of direct comparison of stroke models in rodents, non-human primates and humans. Here, comparing detection of DNA damage after MCA occlusion in rats and baboons, distinct temporal, spatial and quantitative differences were detectable in the corpus striatum [124]. Neuronal cell death in the primates occurred more rapidly than in rats, being 20 times higher already after 2 h but was nearly identical at 20 h. It is suggested that the baboon data more likely reflect the situation in humans, but clear evidence is lacking. More unsettling is the situation when looking at neuroprotective trials. Lysis of an occluded vessel, the first and only stroke therapy successfully translated from an animal model into patients, was adapted from a rabbit model of stroke [140]. On the other hand, NXY-059, which exhibited promising neuroprotective effects in rodent stroke models, was positively replicated in the common marmoset [86] according to the advice of STAIR before going to clinical trials. However, when it came to clinical studies, it failed to show a neuroprotective effect [117].

Nevertheless, it is also clear that ischemic brain injury in non-human primate species results in a clinical syndrome that more equally reflects post-stroke deficits in humans. Currently, major efforts are undertaken to transfer mouse transgenic techniques to non-human primates (for review, see [59]). New genome editing tools such as the CRISPR/Cas9 system [63] will principally enable researchers in the near future to manipulate gene expression cell-type specifically in non-human primates. Given the much higher similarity to humans in brain structure and function compared to rodent animal models, one may expect a deeper understanding of the pathophysiology of stroke—and, much more importantly, will be able to translate this knowledge into effective therapies of ischemic stroke in both the acute and chronic state. Apart from the highly expensive housing of these nonhuman primates, the major issue will be whether society will go along and accept the idea that these experiments are ethically acceptable. The various aspects of ethical issues are critically reviewed by Cook and Tymianski [20].

## Animal models of post-stroke recovery and long-term post-stroke complications

Although mechanisms of spontaneous recovery after stroke have been studied in both rodents and humans for a long time [27, 115], the frustrating translational failure of neuroprotective strategies in acute stroke gave new impetus toward studies on neuroregeneration. The challenges in models of post-stroke recovery differ from those in acute stroke models. Well-standardized lesions in defined brain areas are necessary to record the plasticity processes. Although principally the complete repertoire of stroke models can be used, the more suitable ones go along with low mortality and the possibility to produce small and standardized lesions. The peri-infarct cortex, closely connected ipsilateral areas but also contralateral and remote regions are of particular importance. Therefore, in particular the photothrombosis model and the endothelin-1 model have been used to analyze recovery processes. Although the principal events leading to post-stroke recovery are widely concordant in humans and animals, the kinetics differ considerably. While recovery processes in rodents are almost completed 4 weeks post-stroke, in humans they primarily occur within about 3 months but may even continue for years (for review, see [17, 138]). The exact reason for this difference is not known up so far [74].

Of utmost importance is the use of behavioral tests in the long run, which should ideally test for sensorimotor performance, cognitive performance and mood changes. This is also one big difference between experimental models and reality. While multiple studies focus on the first days after stroke, the final degree of disability in human patients typically is measured months to years after the ischemic event. On the other hand, chronic stroke comes along with complications that have also led to the development of specific models. One frequent event in more than one-third of stroke survivors is the development of a post-stroke depression. The biological basis is yet widely unclear. A number of models have developed to specifically cover this problem [75]. Another frequent complication in up to 30% of stroke patients is development of epileptic seizures and a prevalence of post-stroke epilepsy of 2–4%. Spontaneous epilepsy in rats develops in up to 15% with high variability. Specific models for post-stroke epilepsy are rare [100].

## Conclusion

On the one hand, ischemic stroke is principally a simple and well-defined pathological condition with interruption of blood flow and consecutive damage of dependent tissue. One the other hand, however, ischemic stroke is a very complex and heterogeneous disorder because of a multitude of modifying factors such as the duration and severity of ischemia, presence or absence of functioning collateral systems and an adequate systemic blood pressure, etiology and localization of the infarct as well as age, sex, comorbidities with multimedications and genetic background. Strictly speaking, stroke is not a single neurological disease but the manifestation of an underlying systemic problem such as atherosclerosis, inflammation or infection, which may cause infarcts just as well in other organs and manifest, e.g., as a heart attack. Experimental models of ischemic stroke are valuable tools to analyze specific facets of stroke more or less close to human stroke. Being aware of the limitations of the individual models and ideally having support from studies using human tissues are of utmost importance before drawing conclusions concerning stroke in humans. This issue is illustrated by the still persistent failure of successful translation of effective experimental neuroprotective strategies into patients suffering from this devastating disease.
